# Olaparib in the therapy of advanced ovarian cancer: first real world experiences in safety and efficacy from China

**DOI:** 10.1186/s13048-019-0594-1

**Published:** 2019-11-28

**Authors:** Jing Ni, Xianzhong Cheng, Rui Zhou, Xia Xu, Wenwen Guo, Xiaoxiang Chen

**Affiliations:** 1 Department of Gynecologic Oncology, The Affiliated Cancer Hospital of Nanjing Medical University, Jiangsu Cancer Hospital, Jiangsu Institute of Cancer Research, 42# Baiziting Street, Nanjing, Jiangsu 210009 People’s Republic of China; 2 Department of Chemotherapy, Nanjing Medical University Affiliated Cancer Hospital, Jiangsu Cancer Hospital, Jiangsu Institute of Cancer Research, 42# Baiziting Street, Nanjing, Jiangsu 210009 People’s Republic of China; 3 Department of Pathology, The Second Affiliated Hospital of Nanjing Medical University, Nanjing, Jiangsu 210009 People’s Republic of China

**Keywords:** Olaparib, Ovarian cancer, Safety;short-term efficacy

## Abstract

**Purpose:**

Poly (ADP-ribose) polymerase (PARP) inhibitor, is a milestone in treatment of ovarian cancer. However, there is no real world study from China regarding the clinical outcome of the taking PARP inhibitor (PARPi), Olaparib(Lynparza™). The goal of this research is to evaluate the side effects and short-term efficacy in advanced ovarian cancer patients who administered Olaparib.

**Methods:**

Patients with ovarian cancer, fallopian tube cancer and peritoneal cancer that treated with Olaparib in The Affiliated Cancer Hospital of Nanjing Medical University between September 2018 and June 2019 were recruited. The drug associated Adverse Events (AEs) were collected and short-term efficacy were analyzed by modified Response Evaluation Criteria in Solid Tumors (mRECIST) .

**Results:**

Of all 28 enrolled patients, 92.9% were ovarian cancer, 7.1% were fallopian tube cancer, and 39.3% cases harbored germline BRCA-mutation. There were 6(21.4%) patients received Olaparib after multi-line chemotherapy, and 10 patients (35.7%) as second-line maintenance therapy and 2 patients (7.1%) as first-line maintenance therapy. There were still other 10 cases (35.7%) received Olaparib as exploratory therapy. Abdominal distention, decreased blood pressure, increased body hair, thirsty, burning sensation of stomach and leg swelling were newly reported AEs. Serious Adverse Events(SAEs) were usually managed by dose interruption or dose reduction, rather than discontinuation. 3 patients discontinued treatment, 8 patients received reduced dose of Olaparib, and 4 patients stopped therapy after the alleviation of AEs. Of all 28 enrolled cases, in monotherapy group, 1 of 6 patients achieved stable disease(SD) and also 2 patients achieved stable disease(SD) combined with anti-angiogenic drugs when disease progressed. 2 patients achieved complete remission(CR) and 3 patients were stable with exploratory therapy.

**Conclusions:**

The AEs of Olaparib were all manageable. For the first time, we also identified several AEs such as abdominal distention, decreased blood pressure, increased body hair, thirsty, burning sensation of stomach and leg swelling during the follow-up which have not been reported. The short-term efficacy was observed in some exploratory cases that provided new potential indication to PARPi-related clinical trials.

## Background

Ovarian cancer accounts for about 4% of cancer deaths among women worldwide, and is the most lethal gynecological malignancy [1]. In 2019, it is estimated that there will be approximately 22,530 cases of new identified ovarian cancer, and more than 13,980 women will die from it in the United States [2]. The number of new cases of ovarian cancer in China reached 52,100 in 2015, of which about 22,500 died [3]. The vast majority (> 90%) of ovarian malignancies are epithelial ovarian cancer (EOC), and most patients are diagnosed as FIGO III/IV. The 5-year survival rate of ovarian cancer is about 30%. Currently, the standard treatment for advanced epithelial ovarian cancer is maximal cytoreductive surgery and platinum-based chemotherapy [4].

Although the majority of patients with ovarian cancer can benefit from the first-line platinum-based chemotherapy, about 80% of patients will relapse within 1 to 2 years and suffer multiple recurrences, and patients gradually develop into platinum resistance ovarian cancer [5]. Therefore, it is a burning issue to extend progression-free period and thus improve the 5-year survival rate.

Poly adenosine diphosphate ribosome polymerase (PARP) is a DNA repair enzyme that plays a key role during DNA repair. PARP is activated when DNA is damaged and broken. As a receptor of DNA damage, PARP can recognize and bind to where DNA breaks, and then activate and catalyze the ribosylation of receptor proteins. PARPi inhibits the repair processes of DNA single-strand damage that can be transferred to double-strand damage (DSB) during the formation of DNA replication fork. Also, DSB can be repaired by homologous recombination (HR) pathway. When homologous recombination repair defects present in tumor cells (such as BRCA1 and BRCA2 mutations) that make DSB damage unrepairable, PARP inhibitors and homologous recombination repair defects react in the lethal synthesis of tumor cells [6].

Olaparib (Lynparza™) is the first-in-class oral PARPi. Previous studies have indicated that ovarian cancer patients with germline BRCA mutations platinum-resistant to multi-line chemotherapy could be benefit from Olaparib monotherapy with median progression-free survival (PFS) 7 months and overall survival(OS)16.6 months [7]. Both Study 19 and SOLO2 showed that Olaparib maintenance therapy significantly increased PFS without any detrimental effect on quality of life for those patients with no BRCA-mutated or BRCA-mutated platinum-sensitive recurrent serous ovarian cancer respectively [8, 9]. Further, the favorable results of SOLO1 showed that Olaparib provided a substantial clinical benefit among women in newly diagnosed advanced ovarian cancer with a BRCA1/2 mutation [10]. Based on above mentioned clinical trials, new models and methods for the treatment of ovarian cancer are introduced.

Currently, Olaparib is approved for maintenance treatment of platinum-sensitive relapsed ovarian cancer in many regions, such as the United States, Europe and China [10]. Moreover, it can still be used for maintenance treatment of BRCA mutation after remission of first-line platinum chemotherapy in the United States, Europe and Japan, for monotherapy of germline mutation after third-line chemotherapy only in the United States [11]. Up to present, there is no relevant report of real world data on the administration of Olaparib from China. In this study, the adverse events and short-term effects of Olaparib for patients with ovarian cancer in the real world were retrospectively analyzed.

## Materials and methods

### Study population

Present study was approved by the ethics committee of The Affiliated Cancer Hospital of Nanjing Medical University. Informed consent was obtained from all involved participants. Patients with ovarian cancer, fallopian tube cancer or primary peritoneal cancer that were treated with Olaparib (Lynparza™) between September 2018 to June 2019 at our cancer center were enrolled. All patients took Olaparib for more than 28 days and were followed up with CA125 or imaging examination. We recorded the basic characteristics of these patients, including the age, Eastern Cooperative Oncology Group performance status (ECOG PS) before the start of the treatment, histological type of the primary lesion, history, clinical stage on the basis of Federation International of Gynecology and Obstetrics (FIGO), BRCA mutation, history of therapy before and after the using of Olaparib and the follow-up. Safety was monitored by recording patients, chief complaint, physical examinations, vital signs, adverse events, as well as hematology and clinical chemistry tests.

### Group standard

According to the National Comprehensive Cancer Network (NCCN) 2019.V1 guidelines for ovarian cancer, patients who progresses during initial treatment, or completely alleviates after initial treatment (cytoreductive surgery and platinum-based chemotherapy), but recur within 6 months are defined as platinum-resistant ovarian cancer. Patients who relapse more than 6 months are considered as platinum-sensitive ovarian cancer. Furthermore, patients were divided into first-line maintenance treatment group, second-line maintenance treatment group and monotherapy after multi-line(≥3 lines) treatment group in accordance with indications approved by Food and Drug Administration (FDA). The rest of patients that were not applied in the scope of indications were classified as exploratory therapy group.

### Drug administration

Initially, all patients were orally given the standard dose of 300 mg Olaparib twice a day, and most patients were discontinued upon the progressive disease (PD) or intolerable adverse reaction. A few patients were treated with anti-angiogenic drugs as the disease progressed. Adverse events were graded according to National Cancer Institute Common Terminology Criteria for Adverse Events (NCI CTCAE) version 4.0. There were 8 patients who reduced the dose of Olaparib after the evaluation of adverse events, and 4 patients with severe hematological adverse events continued Olaparib treatment after relief.

### Statistical analysis

Descriptive statistics of clinical and demographic characteristics were summarized. Safety analysis was performed among all enrolled patients who received greater than or equal one dose. AEs were graded according to NCI CTCAE version 4.0. The baseline of CA125 was used as the reference value, and all data were converted to natural logarithm. Short-term efficacy was classified as CR, partial remission (PR), SD and PD by modified Response Evaluation Criteria in Solid Tumors version 1.1(RECIST 1.1).

## Results

### Patients’ characteristics

The average age of the 28 included patients was 59 years (range 36–79), and 92.9% were ovarian cancer, 7.1% were diagnosed as fallopian tube cancer. The ECOG scores of all patients were 0–1. Advanced ovarian cancer, known as FIGO III or IV, affected 19 (67.9%) and 3 (10.7%) of patients, respectively. 25 patients suffered from high-grade serous adenocarcinoma, and the remaining 3 cases were endometrioid carcinoma, mixed serous and endometrioid carcinoma and mixed serous and mucious carcinoma. 39.3% patients harbored BRCA 1/2 mutation, also 39.3% patients had BRCA wild type. Baseline characteristics of eligible patients were summarized in Table 1. The family history of the patients was classified according to their BRCA status (Table 2).

**Table 1:** Baseline characteristics in 28 patients. Values are reported as frequency (n [%]) or as mean (range)

Characteristic	Number of patients (percent)
Age, yrs	
≤ 59	13 (46.4)
> 59	15 (53.6)
Primary tumor location	
Ovary	26 (92.9)
Fallopian tube	2 (7.1)
Peritoneum	0 (0)
International FIGO stage	
I	4 (14.3)
II	2 (7.1)
III	19 (67.9)
IV	3 (10.7)
Histological type	
Serous	25 (89.3)
Endometrioid	1 (3.6)
Mixed serous and endometrioid	1 (3.6)
Mixed serous and mucious	1 (3.6)
Family history of cancer	
Yes	10 (35.7)
No	15 (53.6)
Missing data	3 (10.7)
ECOG	
0	18 (64.3)
1	10 (35.7)
BRCA	
BRCA 1/2 mutation	11 (39.3)
Wild type	11 (39.3)
Unknown	6 (21.4)
Sensitivity to platinum-based chemotherapy	
Platinum-sensitive	11 (39.3)
Platinum-resistant	13 (46.4)
Unknown	4 (14.3)
Categories of therapy	
First-line maintenance therapy	2 (7.1)
Second-line maintenance therapy	10 (35.7)
Treatment after multi-line chemotherapy	6 (21.4)
Exploratory therapy	10 (35.7)
Primary cytoreductive surgery	
Yes	27 (96.4)
No	1 (3.6)
Secondary cytoreductive surgery	
Yes	6 (21.4)
No	22 (78.6)
Combination of anti-angiogenic agents	
Yes	4 (14.3)
No	24 (85.7)

Abbreviations: FIGO, International Federation of Gynecology and Obstetrics; ECOG, Eastern Cooperative Oncology Group

**Table 2:** Classification of family history according to BRCA status

BRCA status	Family history of cancer
BRCA unknown	Skin cancer, leukemia
BRCA1m	Ovarian cancer, pancreatic cancer, gastric cancer, cervical cancer, esophageal cancer
BRCA2m	Ovarian cancer, lung cancer, rectal cancer, retroperitoneal neoplasms
BRCAw	Gastric cancer, lymphoma, gingival cancer, breast cancer, rectal cancer

### Patients’ classification

In this study, there were 11 patients diagnosed with platinum sensitive ovarian cancer, 13 patients with platinum resistance ovarian cancer, and 4 patients with unknown platinum reaction. Subsequently, we divided the enrolled patients into 4 groups. Among them, 2 patients received first-line maintenance treatment, 10 received second-line maintenance treatment, 6 received single-drug treatment after third-line chemotherapy, and 10 received exploratory drugs, 6 of whom were patients with BRCAwt and BRCA unknown after multi-line(≥3 lines) treatment. The other three patients harbored BRCAm were the first recurrence platinum sensitivity ovarian cancer or fallopian tube cancer, one of them recurred after maximal cytoreductive surgery and platinum-based chemotherapy, the two patients recurred after only maximal cytoreductive surgery without postoperative chemotherapy. The last patient was treated with Olaparib after only chemotherapy without surgery. In monotherapy group, four platinum-resistant patients harbored gBRCA mutations after multi-line chemotherapy used Olaparib. One of the other two platinum-resistant patients in monotherapy group carried the suspected pathogenic frameshift mutation c.7198 Inst (p.Gly2400Valfs*12p), and the other one carried the unknown missense mutation c.112 g > A (p.Glu38Lys), both of which were possible pathological mutations. Anti-angiogenic agents were added to 4 patients with the progression of the disease.

### Drug related AEs

The most common all grade adverse events were fatigue or asthenia (60.70%), decreased appetite (42.9%), anemia (42.9%), nausea (39.3%), arthralgia (32.1%) and vomiting (25%). Most patients had grade 1 or 2 AEs. Adverse reactions of grade 3 or 4 were observed in few cases. Anemia, abdominal pain and thrombocytopenia occurred in three patients, two patients and one patient, respectively. Other AEs included diarrhea (10.7%), constipation (17.9%), dysgeusia (17.9%), dizziness (10.7%), cough (10.7%), back pain (14.3%) and dyspnea (3.6%). These AEs occurred within 3 months after administration.

We also identified several AEs that have never been reported previously, including abdominal distention (14.3%), decreased blood pressure (3.6%), increased body hair (3.6%), thirsty (3.6%), burning sensation of stomach (3.6%) and leg swelling (3.6%). The occurrence time of the newly AEs was similar to that of the known AEs. Similarly, most patients had mild AEs. Only 1 patient with declined blood pressure stopped Olaparib treatment.

AEs were usually managed by dose interruption or dose reduction, rather than discontinuation. 3 patients discontinued treatment, 8 patients received reduced treatment, and 4 patients interrupted treatment after the alleviation of AEs (Table 3). Short-term efficacy was not affected among 2 of 8 patients who took low dose of Olaparib. Further, the short-term clinical outcome was not affected for 3 of the 4 patients who orally received 150 mg of Olaparib twice a day. After they were recovered from the AEs, these three patients all received reduced dose of Olaparib. Over the following 3 months, the levels of CA125 were elevated but the imaging showed no recurrence among 2 out 3 patients.

**Table 3:** Summary of Adverse Events

Adverse Event	Any Grade	Grade 3 or 4
	number of patients (percent)	
Fatigue or asthenia	17 (60.7)	0 (0)
Anemia	12 (42.9)	3 (10.7)
Decreased appetite	12 (42.9)	0 (0)
Nausea	11 (39.3)	0 (0)
Arthralgia	9 (32.1)	0 (0)
Vomiting	7 (25.0)	0 (0)
Abdominal pain	5 (17.9)	2 (7.1)
Constipation	5 (17.9)	0 (0)
Dysgeusia	5 (17.9)	0 (0)
Neutropenia	5 (17.9)	0 (0)
Thrombocytopenia	4 (14.3)	1 (3.6)
Back pain	4 (14.3)	0 (0)
Diarrhea	3 (10.7)	0 (0)
Dizziness	3 (10.7)	0 (0)
Upper abdominal pain	3 (10.7)	0 (0)
Cough	3 (10.7)	0 (0)
Dyspnea	1 (3.6)	0 (0)
Headache	0 (0)	0 (0)
Dyspepsia	0 (0)	0 (0)
Newly observed		
Abdominal distention	4 (14.3)	0 (0)
Decreased blood pressure	1 (3.6)	0 (0)
Skin rash	1 (3.6)	0 (0)
Increased body hair	1 (3.6)	0 (0)
Thirsty	1 (3.6)	0 (0)
Burning sensation of stomach	1 (3.6)	0 (0)
Leg swelling	1 (3.6)	0 (0)
Led to discontinuation of intervention	3 (10.7)	–
Led to dose reduction	8 (28.6)	–
Led to dose interruption	4 (14.3)	–

Adverse events were graded according to National Cancer Institute Common Terminology Criteria for Adverse Events(NCI CTCAE), version 4.0

The most common AEs that led to interruption were anemia and decreased blood pressure. 2 of 3 patients who discontinued treatment had severe anemia and 1 also had severe leucopenia. The major reasons for patients who reduced the amount of Olaparib were digestive tract reactions, bone marrow suppression including anemia, leucopenia and thrombocytopenia and abdominal pain.

### Short-term efficacy

In exploratory therapy group, 2 patients achieved CR, 3 patients achieved SD, whereas 5 patients had disease progressed. One patient took Olaparib when she firstly recurred after merely maximal cytoreductive surgery without postoperative chemotherapy and another patient was treated with Olaparib after only chemotherapy without surgery. These two patients with CR were followed up for 3 months and 4 months, respectively (Table 4). Three patients with SD were followed up for 6 months, 7 months and 2 months, respectively. The first patient used Olaparib also when she recurred after maximal cytoreductive surgery and platinum-based chemotherapy, the second patient was treated with Olaparib when she relapsed after only maximal cytoreductive surgery without postoperative chemotherapy and the third platinum-resistant recurrent ovarian cancer patient with BRCAw took Olaparib after multi-line therapy.

**Table 4:** Short-term efficacy of exploratory therapy

Short-term efficacy	Number of patients (Percent)
Complete response (CR)	2 (20.0)
Partial response (PR)	0 (0)
Stable disease (SD)	3 (30.0)
Progressive disease (PD)	5 (50.0)

Short-term efficacy was classified by modified Response Evaluation Criteria in Solid Tumors version 1.1(RECIST 1.1)

In the multi-line therapy group, one patient was assessed as SD after monotherapy for 3 months (Table 5). Three platinum-resistant recurrent ovarian cancer patients added anti-angiogenic agents (Cediranib was purchased by herself from abroad or Apatinib was produced in Jiangsu HengRui Pharmaceutical co. LTD) after progressed, 2 patients fortunately achieved SD in seven months and three months, respectively (Table 6).

**Table 5:** Short-term efficacy of monotherapy after multi-line chemotherapy

Short-term efficacy	Number of patients (Percent)
Complete response (CR)	0 (0)
Partial response (PR)	0 (0)
Stable disease (SD)	1 (33.3)
Progressive disease (PD)	2 (66.7)

**Table 6:** Short-term efficacy of monotherapy combined with anti-angiogenic agents after PD with monotherapy

Short-term efficacy	Number of patients (Percent)
Complete response (CR)	0 (0)
Partial response (PR)	0 (0)
Stable disease (SD)	2 (66.7)
Progressive disease (PD)	1 (33.3)

Another patient found a continuous increase in CA125 level during the second-line maintenance treatment, and a persistence decrease in CA125 level occurred when Apatinib was added under the strong personal will of herself.

**Fig. 1:**
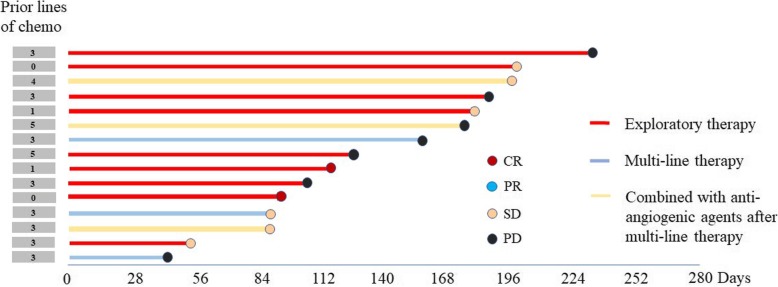
Water plot of best response by RECIST1.1. Note: This is a water plot of the latest imaging evaluation in multi-line therapy group and exploratory therapy group, including 6 patients in multi-line therapy group and 10 patients in exploratory therapy group. Three patients in multi-line treatment group added anti-angiogenic drugs when developed progressive disease, two of them achieved stable disease after combined two drugs. In exploratory therapy group one platinum-resistant ovarian cancer patient with non-BRCA mutation achieved progressive disease after using Olaparib for one month and she died soon, which was not shown in the figure. Finally, the short-term efficacy of 9 patients in exploratory therapy group were shown in the figure

**Fig. 2:**
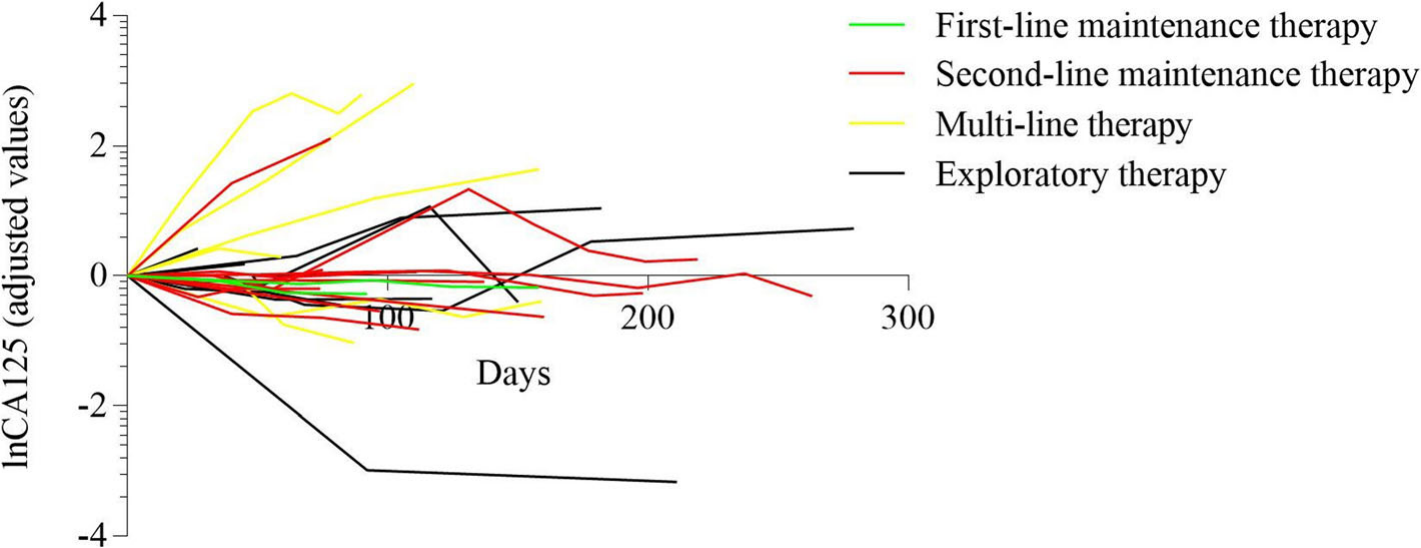
CA125 values in each group. Note: The CA125 level of the first follow-up was used as the reference value, and all data were converted to natural logarithm. CA125 follow-up data were obtained from 2 patient in the first-line maintenance treatment group, 10 patients in the second-line maintenance treatment group, 6 patients in the multi-line therapy group, and 10 patients in the exploratory therapy group

**Fig. 3:**
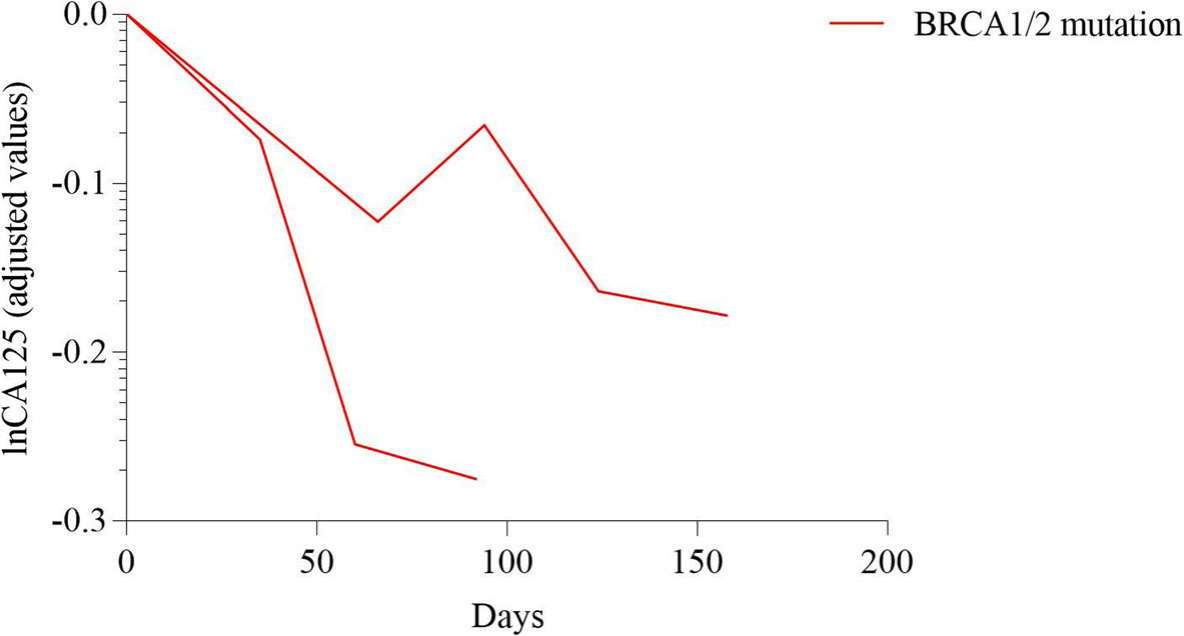
CA125 in patients with different BRCA status in the first-line maintenance group. Note: Two patients with BRCA mutation in the first-line maintenance treatment group was followed up with CA125

**Fig. 4:**
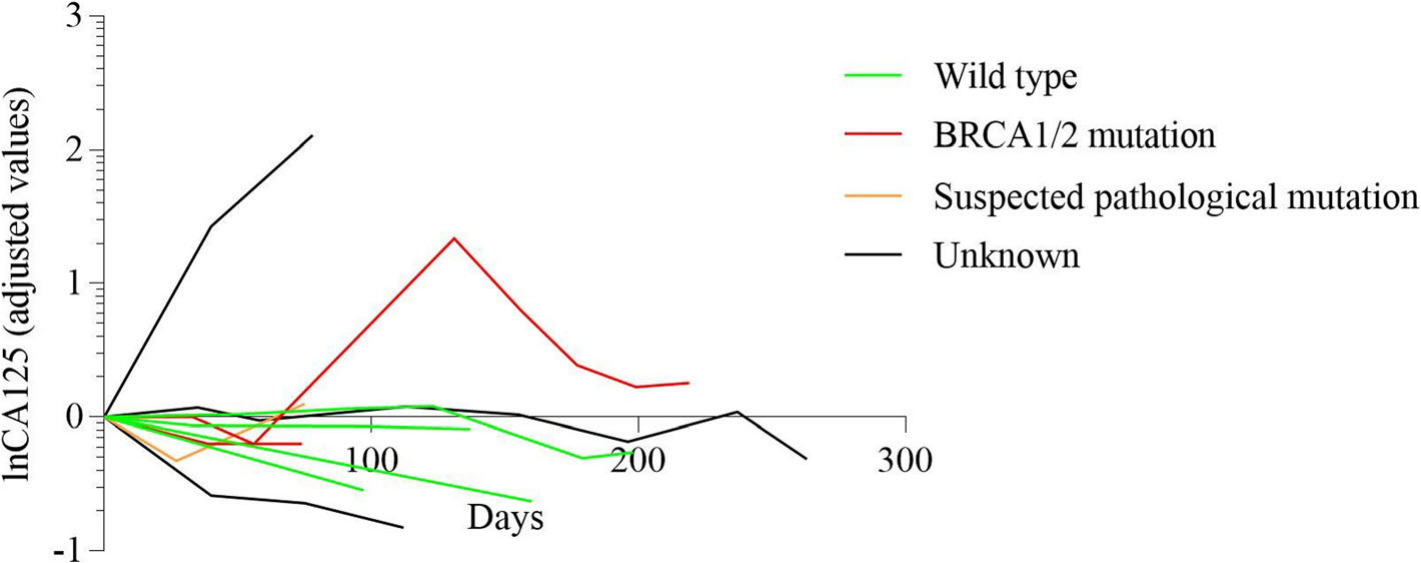
CA125 in patients with different BRCA status in the second-line maintenance group. Note: There were two patients with BRCA mutation, four patients with BRCA wild-type, one patient with BRCA suspected pathological mutation and also three patients with BRCA status unknown in this group

**Fig. 5:**
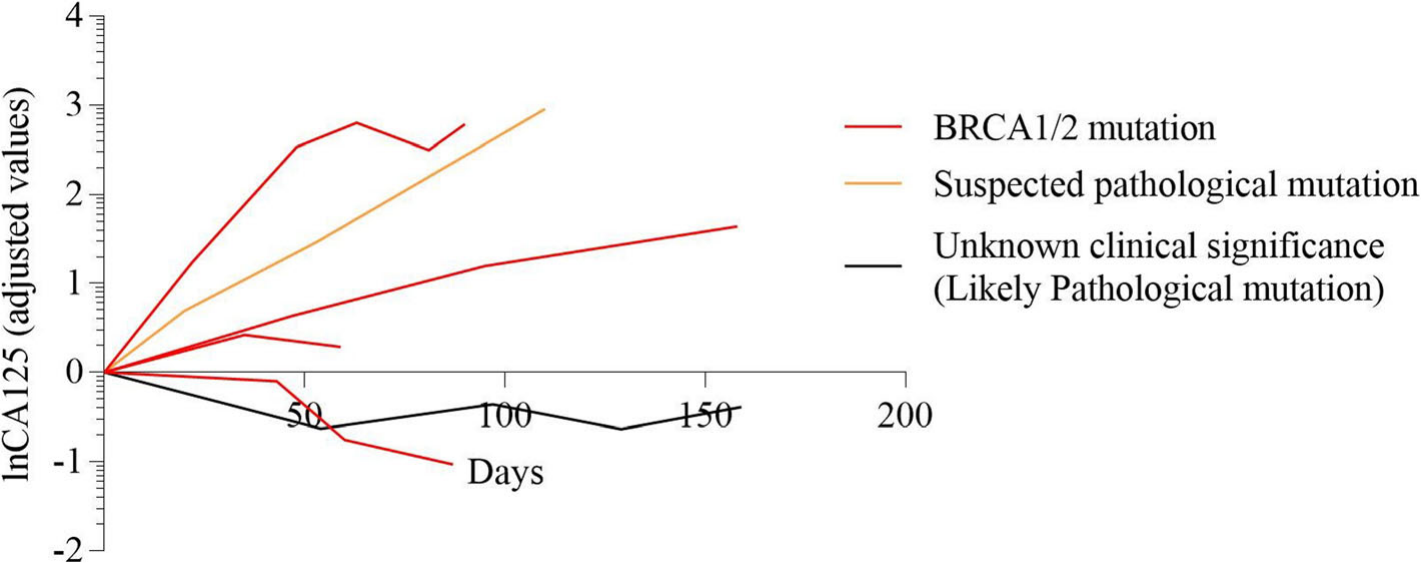
CA125 in patients with different BRCA status in multi-line therapy group. Note: There were four patients with BRCA mutant, one patient with BRCA suspected pathological mutation and also one patient with BRCA unknown clinical significance mutation in this group

**Fig. 6:**
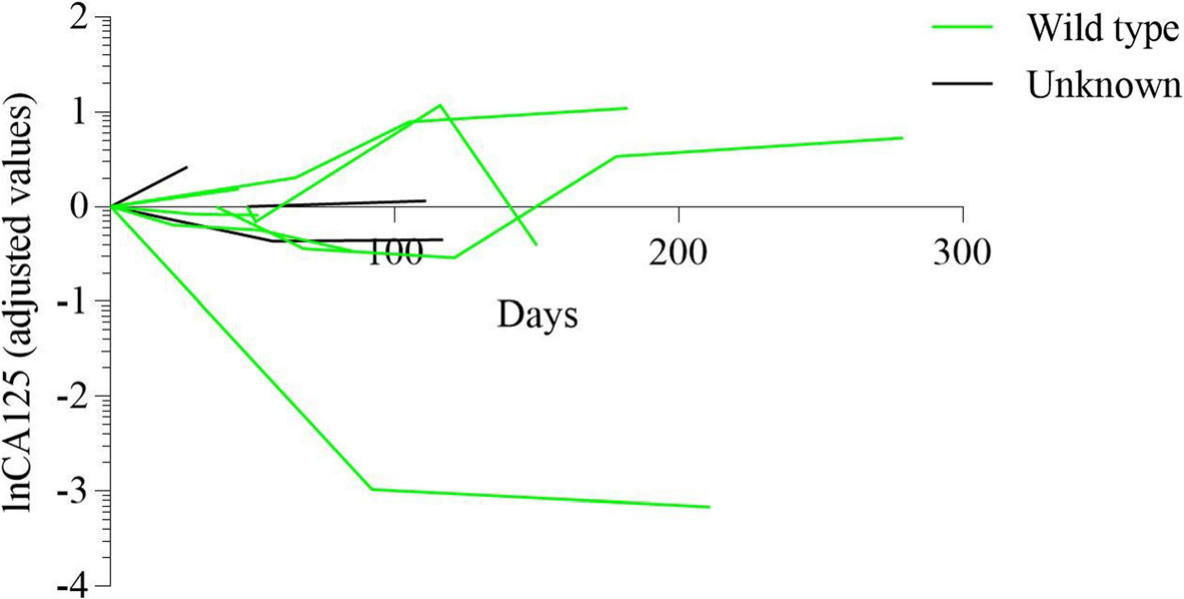
CA125 in patients with different BRCA status in exploratory therapy group. Note: There were seven patients with BRCA wild-type and three patients with BRCA status unknown in this group

The imaging evaluation of multi-line therapy group and exploratory therapy group on the basis of RECIST 1.1 were shown in Fig. 1. Meanwhile, CA125 level were measured among various groups with different BRCA mutation status in Figs. 2, 3, 4, 5 and 6. The levels of CA125 in the first-line maintenance and the second-line maintenance groups were comparatively lower than that in the other two groups.

## Disscusion

Molecular targeted therapy is currently the central issue of cancer treatment. Targeted therapy for non-small cell lung cancer has become one of well-accepted treatment principles in the worldwide [12]. Ovarian cancer is the most fatal gynecologic malignant tumor. PARP inhibitors have revolutionized the traditional treatment strategy of ovarian cancer. The results of two phase III trials, SOLO1 and SOLO2, showed that the Olaparib as the first-line/second-line maintenance therapy could significantly improve the progression-free survival and reduce the risk of disease recurrence and mortality [9, 10]. Also, in previous study, it was also found that Olaparib monotherapy after the multi-line treatment in patients with germline BRCA mutations could increase the tumor response rate [7]. SOLO3 was reported in 2019 ASCO, suggesting that that multi-line platinum-sensitive patients with germline mutations could be significantly benefit from Olaparib (ORR72%, PFS 13.2 m vs. 8.5 m) [13]. The study of CLIO indicated that the efficiency of multi-line platinum-resistant patients was 18%, which is superior to 6% of standard chemotherapy [14]. These latest findings further validated that PARP inhibitors could offer a non-chemotherapeutic treatment option for platinum-sensitive or platinum-resistant relapse patients. EVOLVE study also showed that the addition of cediranib treatment reached the expected efficacy for patients with PARPi resistance [15].

Among the four groups in this study, all patients had AEs, and the most common adverse reaction were fatigue, loss of appetite, anemia, nausea and vomiting, and the majority patients had grade 1–2 AEs. Seventeen percentage of patients developed grade 3–4 AEs, including severe anemia, abdominal pain, and thrombocytopenia. AEs were usually managed by dose interruption or dose reduction, rather than discontinuation. In our study, the median time of occurrence of AEs and SAEs was consistent with previous studies and SOLO1 from China cohort [16]. However, the incidence of serious adverse reactions was lower, which may be due to the relatively small number of subjects in our study. For the first time, we also identified a few AEs during the follow-up which have not been reported. A platinum-sensitive recurrence patient had a substantial reduction of blood pressure after 4 months of Olaparib monotherapy. The patient’s blood pressure went back up when she was given in half of the standardized dosage. The other mild AEs first identified in our study also included abdominal distention, increased body hair, thirsty, burning sensation of stomach and leg swelling. Interestingly, all patients with these unreported AEs were found in patients harbored BRCA mutations, and whether such BRCA mutations played a role in AEs the occurrence of AEs remains to be explored.

A multicentre, single-arm, phase 2 study that evaluated the safety and activity of Niraparib in patients with relapsed ovarian cancer who were treated with three or more previous chemotherapy regimens. Patients were orally received 300 mg of Niraparib once per day. Since the patients showed reduced platelet, the dose was adjusted from 300 mg to 200 mg. AEs were significantly reduced, and the efficacy of Niraparib was not affected by AEs [17]. Similarly, we also found that some patients who received oral reduction dose of Olaparib did not affect the short-term effect in our cases. It is suggested that Chinese people or population with a baseline BMI or baseline AUC who received lower dose of Olaparib could be benefit from it and reduced the incidence of treatment-emergent adverse events.

We also analyzed the short-term efficacy of the posterior monotherapy group and exploratory treatment group, and found that 1 patient in the posterior monotherapy group achieved stability. 2 patients with BRCA mutant in this group progressed who achieved stable disease after adding anti-angiogenic drugs. 1 patient was platinum-sensitive recurrent ovarian cancer and the other was platinum-resistant recurrent ovarian cancer, which were consistent with the results of EVOLVE [15]. Interestingly, in the exploratory treatment group, we found that one patient achieved CR after using Olaparib when she firstly recurred after merely maximal cytoreductive surgery without postoperative chemotherapy and another patient also achieved CR after using Olaparib when she only took chemotherapy without suffering surgery. The benefits of these exploratory drugs provided us with new evidence for clinical trials in the future.

BRCA mutations that are closely related to the potency of PARP inhibitors under some circumstances. Previous studies have shown that the risk of ovarian cancer in the general population is 1–2%, while the risk of population with BRCA1 mutant and BRCA2 mutant are 39–63% and 16.5–27%, respectively [18].BRCA mutant are closely associated with breast cancer and may be also related to prostate cancer [19], pancreatic cancer [20], and cutaneous melanoma [21]. In this study, family history of cancer for patients was also considered. It was found that the family with BRCA1 mutant had pancreatic cancer, gastric cancer, cervical cancer and esophageal cancer, while the family with BRCA2 mutant had lung cancer, rectal cancer and close relatives of retroperitoneal tumor. The results suggest that BRCA mutant may be associated with tumorigenesis in a variety of cancers. And further research is needed to confirm the heredity in population with BRCA mutant. Based on the clinical benefits of PARP inhibitors in ovarian and pancreatic cancer with BRCA mutant [22], whether the indications for PARP inhibitors for tumors associated with BRCA mutations should be further explored.

## Conclusion

In conclusion, our study evaluated the side effects and short-term efficacy in ovarian cancer patients who were treated with Olaparib, in the real-world setting. This study mainly focused on AEs and SAEs of patients that are consistent with the previously published results. Nevertheless, we also observed several unreported AEs. In addition, we are concerned about the clinical efficacy of Olaparib, although there is a certain clinical remission rate within short time, but the clinical outcome for these patients with ovarian cancer needs to be further followed up. Also, the benefits in exploratory treatment group provided new potential indication to PARPi-related clinical trials.

## Data Availability

We would not share the data and material used in this manuscript, because we need them for further research.

## Abbreviations

AE: Adverse Events
EOC: Epithelial Ovarian Cancer
PARP: Poly Adenosine diphosphate Ribosome Polymerase
RECIST: Response Evaluation Criteria in Solid Tumors
SAE: Serious Adverse Events
